# Molecular docking analysis of proflavin with the Wnt pathway targets for OSCC

**DOI:** 10.6026/97320630019464

**Published:** 2023-04-30

**Authors:** Jayanthi Pazhani, Vishnu Priya Veeraraghavan, Selvaraj Jayaraman

**Affiliations:** 1 Centre of Molecular Medicine and Diagnostics (COMManD), Department of Biochemistry, Saveetha Dental College and Hospitals, Saveetha Institute of Medical and Technical Sciences, Saveetha University, Chennai-600077, India

**Keywords:** Oral carcinoma, wnt signaling, proflavine, molecular docking

## Abstract

Wnt signaling pathway plays a critical role in tumor progression and metastasis of various cancer types. Therefore, it is of interest to document the molecular docking analysis of proflavin with the Wnt pathway targets (GSK3β, β-catenin, and VIM) for oral squamous cell carcinoma (OSCC). These results suggest that targeting the wnt signaling downstream targets with proflavine might leads to the better outcome for therapeutic outcome for the inhibition of invasion and metastasis in oral squamous cell carcinoma.

## Background:

Oral Squamous Cell carcinoma (OSCC) is one of the common cancers around worldwide. In particular, it is a major health threat in the Indian subcontinent, where it ranks among the top three types of cancer in the nation [1]. An age-adjusted rate of oral cancer in India is high, which is, 20 per 100,000 population and accounts for over 30% of all cancers in the country [2]. In India, as of cultural, ethnic, geographic factors, and the popularity of addictive habits, the occurrence of oral cancer was intense. Moreover, oral cancer is generally occurred due to several factors like tobacco and tobacco related products, alcohol, genetic disposition, and hormonal factors which that are suspected as most possible common factors [3]. Regardless of availability of newer diagnostic and therapeutic strategies concerning oral squamous cell carcinoma (OSCC) diagnosis and therapy, the survival rate of patients has not shown much improvement [3, 4]. Mostly, tumor progression claims from an aberrant communication between the cancer cells and their micro environmental conditions. The microenvironment involves of host cells, which in associated with the cancer cells, that produces the extracellular matrix (ECM), proteinases, and cytokines [5]. The complex which the cellular tumor stroma includes is the immuno competent and inflammatory cells, endothelial cells, fibroblasts, and a subtype specific to fibroblasts known as myofibroblasts [6]. However, the cancer cell factors acting upon the microenvironment, the host factors sent to the cancer cells, and their intracellular signalling pathways are largely unknown [6, 7].

Proflavine (3,6-diaminoacridine), an acridine dye is a known for DNA intercalating agent. Proflavine, that associated with a flavine nucleus that can penetrate the epidermal and dermal structures in the *in-vivo* stained cells and also accumulated in the cell nuclei, only cells of the central nervous system did not engage to any proflavine [7]. The known drug, proflavine mainly use as a cytological tool as for it was not wide-spread in contempt its various advantages. And also, it is capable to intercalate DNA which has provided many applications including anti-cancer, anti-bacterial, and anti-viral drugs [8]. However, the efficacy of proflavine for targeting the epithelial to mesenchymal transition through signaling pathway in oral squamous cell carcinoma remains poorly understood [9].

Wnt signaling is mainly used as regulator for diverse biological processes and functions, that including the embryonic development, tissue regeneration, haematopoiesis, cell survival, cell proliferation, and differentiation [9, 10]. Wnt signaling pathway is vital for multiple stages of tooth development and it plays a crucial role for dental epithelial cell proliferation and differentiation [10]. Moreover, it has been efficient that functional dysregulation of the Wnt signaling pathway can promote oral cancer development and progression [11]. The deregulation of the Wnt signaling pathway is significantly associated with prognosis in patients with OSCC. Therefore, elucidation of the molecular mechanisms regulating the initiation and progression of oral tumorigenesis will provide insight into the etiologic underlying the diseases. By targeting the wnt pathway downstream targets will be a potential to improve therapeutic efficacy, as well as to design more effective treatment strategies for OSCC. Therefore, it is of interest to document the molecular docking analysis [12] of proflavin with the Wnt pathway targets for oral squamous cell carcinoma (OSCC).

## Materials and Methods:

## Protein preparation:

Three-dimensional structures for Wnt signaling targets GSK3β, β-catenin, and vimentin (GSK3β - PDB ID: 1GNG, β-catenin - PDB ID: 1JDH; Vimentin - PDB ID: IGK4) were retrieved from Protein Data Bank (PDB). The .pdb file was submitted to "Build/check/repair model" for correction and "Prepare the PDB file for docking programs" modules where the missing side chains were shown in, a small regularization that was performed. The water positions and the symmetrical alignment were corrected, and also hydrogen molecules were added. Only chain A of the repaired .pdb files were evaluated and conceded to AutodockTools (ADT ver.1.5.6) for. pdbqt file preparation. Moreover, water molecules and non-standard residues were removed, only polar hydrogen was maintained, and Gustier charges were computed for protein atoms by ADT.

##  Ligand preparation:

Proflavine, an acrylamide dye was constructed with ChemSketch-12.01 software and the geometrical representation were optimized using the Austin Model 1 to the corresponding mol2 file that was give in to ADT for pdbqt file preparation and docking with AutoDock4. The geometry of built compound was enhanced; partial charges were also designed, and saved as mol2 files that were passed, as usual, to ADT for pdbqt file preparation.

## Docking Procedure:

Autodock4 (ver. 4.2.6) [12, 13] was employed for docking studies. Lamarckian genetic algorithm with local search (GALS) was used as search engine, with a total of 100 runs. The region of interest, which are generally used by Autodock4 for docking runs and by Autogrid4 for affinity grid maps preparation, was defined in such a way to comprise the whole catalytic binding site by using a grid of 40 x 40 x 40 points with a grid space of 0.325 Å, centres of grid box: x = 23.049; y = 23.526; z = 46.984. Cluster analysis was also performed on the docked results using an RMS tolerance of 2.0 Å. Finally, the more energetically involved cluster poses were evaluated by Python Molecule Viewer (PMV ver.1.5.6) and PyMOL ver.1.1.7 (DeLano Scientific LLC).

## Software used for molecular docking:

The ligand preparation was achieved by using ACD/Chem Sketch 12.01 (Advanced Chemistry Development, Inc), geometrical representations were optimized using Hyperchem 8.0.3 and for protein preparation Wizard of Auto Dock tools 1.5.4 are used for this study. Molecular docking calculation was fully completed using Auto Dock tools and MGL tools 1.5.6 packages (The Scripps Research Institute, Molecular Graphics Laboratory, CA, USA).

## Results and Discussion:

**Figure 1:**
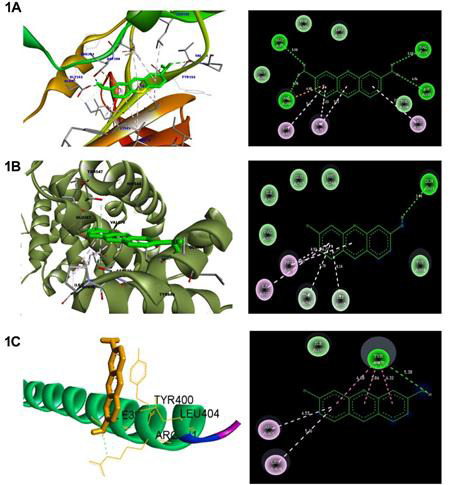
Molecular docking analysis of proflavin with the Wnt pathway targets (A: GSK3β, B:β-catenin, and C: VIM)

Molecular docking is an effective tool to get a optimal position for ligand to bind to a targets active site. These techniques encompass the three dimensional coordinate space of the binding site in the target and that measures the binding interaction of the specific molecules resultant orientation within the binding site forming the complex. The binding affinity values were calculated by the numbers which have maximum magnitude negative.(highest binding affinity or lowest binding energy), Additionally docking were used to conform the drug PROFLAVIN(CID-7099) has anti cancerous activity against the oral squamous cell carcinoma. The drug PROFLAVIN (CID-7099) was associated with wnt pathway targets. The binding energies of the targets GSK3β, CTNBB, VIM has the values of -6.98kcal/mol,-4.99kcl/mol,-3.34kcl/mol. The target proteins highly interacts with the (TYR134, ASP200, LYS85), (VAL570, GLN601),(TYR400,LEU404) these amino acid residues respectively (table:1) . The target GSK3β has the highest affinity when compared with other two targets. The entire compound involved in the tight bounding of hydrogen atoms with the target protein [(Figure 1A), (2.8 Å, N-O, 2.0 Å, O-H) (Figure 1B), (3.0 Å, N-O, 2.1 Å, O-H) (Figure 1C). The curcumin analogue 1-(1,3-benzodioxol-5- yl)-5-(4-hydroxy-3-methoxyphenyl) penta-1, 4-dien-3-one orients in a similar fashion to that of 1-(3,4-dimethoxyphenyl)-5-(4- nitrophenyl) penta-1, 4-dien-3-one and 1-(4-hydroxy-3- methoxyphenyl)-5-(4-methoxyphenyl) penta-1, 4-dien-3-one. However, only one hydrogen bond was observed between the methoxy group and OH of Ser 530 (3.6 Å, O-O, 3.8 Å (O-H). Based on this study, there are three curcumin analogues showed significant inhibition of the enzyme COX-2. It is clear that this compound has the potential to inhibit COX enzymes; however, they need to be confirmed from the biological evaluation and in vitro testing. The 2D interaction was obtained from discovery studio and the 3D structure was obtained from Auto dock vina ver.1.5.4. This work shows that the drug proflavin has the ability to treat the oral squamous cell carcinoma and the future work can be carried out In-vitro and *in-vivo* studies to obtain the overall activity of the drug proflavin for treatments.

## Conclusion:

The molecular docking results suggest a prominent binding affinity between the interaction of proflavine and Wnt signalling targets (GSK3β, β -catenin, and VIM) which are mainly involved in Epithelial-mesenchymal Transition (EMT). Data shows that targeting the wnt signaling pathway targets with a known drug, proflavine could be a better targeting optional for oral squamous cell carcinoma.

## Figures and Tables

**Table 1:** Molecular docking results

**Drug**	**Proteins**	**Kcal/mol**	**Residues**
	GSK3 beta(PDbID: 1GNG)	-6.98	TYR134,ASP200, LYS85
			
Proflavin(CID-7099)	CTNBB (PDB ID:1JDH)	-4.99	VAL570,GLN601
	VIM (PDB ID:1GK4)	-3.34	TYR400,LEU404
			
